# 

**Published:** 1892-01

**Authors:** 


### LITERARY.

We call special attention to our friend, Dr. Kimball's announcement of his new literary, health and dental journal, The Dentist Himself. We are assured that every promise made to his subscribers will be faithfully fulfilled.

An Abstract of the Symptoms, with the latest Dietetic and Medicinal Treatment of Various Diseased Conditions. Part II.—The Physiology of Digestion and Assimilation. Reed & Carnrick, Publishers, 447-9 Greenwich st., New York.

This comprehensive little work will be found of great assistance to the inexperienced practitioner, no less than the older heads, and we doubt not, will meet the expectations of its publishers by filling "a long felt gap in medicinal literature." Guided by the accuracy of the diagnosis of various diseases, therein contained, there will be fewer misconceptions and mistakes in practice, and less useless suffering among patients.

THE SUPREME PASSIONS OF MAN, by Professor Paul Paquin, M. D. The Little Blue Book Co., Battle Creek, Mich., Publishers.

In his introduction the author says: "Several years ago, while making comparative tests of culture media for the nutrition of microbe organisms in the laboratory under my charge at the University of Missouri, I became deeply interested in the striking differences which different food substances produced in a given organism. I found myself capable of altering the shape, size, energies, color, properties, in fact, all the vital attributes, it seemed, merely by varying the kind, quality, and quantity of food material."

The above statement furnishes a comprehensive view of the design, scope and great usefulness of this compact little treatise which gives more information in its 150 pages on subjects relating to moral and physical life, than volumes of less practical works on the same subjects.

THE GLOBE ALMANAC AND 1891 POLITICAL HAND BOOK. St. Paul, Minn. This useful annual of 150 pages, compiled by Messrs. Dodds, Nowell & Black, is a complete, handy reference of last year's more important events and occurrences, arranged with a skill and completeness equal to the best local publications of our leading newspapers, and especially adapted to the North-west.

The authors have our thanks for the following pamphlets, explained by their titles:

EFFECTS OF MASSAGE, compiled by Leroy Henry, Practical Masseur, 2720 Pine st., St. Louis, Mo. A very instructive little work.

A VEGETABLE PLATE, ETC., IN INTESTINAL ANASTOMOSIS, by Professor Robert H. M. Dawbarn, M. D., New York City, illustrated.

THE SOCIAL AND MEDICAL ASPECTS OF INSANITY, by John Prenton, M. D Kansas City, Mo.

